# Alfalfa Root Growth Rate Correlates with Progression of Microtubules during Mitosis and Cytokinesis as Revealed by Environmental Light-Sheet Microscopy

**DOI:** 10.3389/fpls.2017.01870

**Published:** 2017-10-30

**Authors:** Petra Vyplelová, Miroslav Ovečka, Jozef Šamaj

**Affiliations:** Department of Cell Biology, Centre of the Region Haná for Biotechnological and Agricultural Research, Palacký University Olomouc, Olomouc, Czechia

**Keywords:** cell division, developmental imaging, light-sheet microscopy, *Medicago sativa*, microtubules, root growth, transgenic crops

## Abstract

Cell division and expansion are two fundamental biological processes supporting indeterminate root growth and development of plants. Quantitative evaluations of cell divisions related to root growth analyses have been performed in several model crop and non-crop plant species, but not in important legume plant *Medicago sativa*. Light-sheet fluorescence microscopy (LSFM) is an advanced imaging technique widely used in animal developmental biology, providing efficient fast optical sectioning under physiological conditions with considerably reduced phototoxicity and photobleaching. Long-term 4D imaging of living plants offers advantages for developmental cell biology not available in other microscopy approaches. Recently, LSFM was implemented in plant developmental biology studies, however, it is largely restricted to the model plant *Arabidopsis thaliana*. Cellular and subcellular events in crop species and robust plant samples have not been studied by this method yet. Therefore we performed LSFM long-term live imaging of growing root tips of transgenic alfalfa plants expressing the fluorescent molecular marker for the microtubule-binding domain (GFP-MBD), in order to study dynamic patterns of microtubule arrays during mitotic cell division. Quantitative evaluations of cell division progress in the two root tissues (epidermis and cortex) clearly indicate that root growth rate is correlated with duration of cell division in alfalfa roots. Our results favor non-invasive environmental LSFM as one of the most suitable methods for qualitative and quantitative cellular and developmental imaging of living transgenic legume crops.

## Introduction

Plant growth and development is directed by key biological processes establishing proper morphogenetic pattern. Among them, cell division plays an indispensable role. Cell divisions in the root meristem are followed by subsequent cellular expansion in the root elongation zone and ensure sustained development of roots. Plant mitotic cell division is dynamic process controlled by rearrangements of microtubules that are gradually transformed into different microtubule arrays during mitosis and cytokinesis. Before the onset of cell division, cortical microtubules are narrowing to form the preprophase band (PPB) defining the cell division plane (Rasmussen et al., 2011). After nuclear envelope breakdown and the initiation of the mitotic process, microtubules form the mitotic spindle responsible for chromosome partitioning into daughter cells during mitosis (Vos et al., 2004; Marcus et al., 2005; Azimzadeh et al., 2008). This bipolar spindle is formed with its long axis oriented perpendicularly to the PPB plane (Van Damme et al., 2007; Van Damme, 2009). During cytokinesis the bipolar spindle is transformed into the phragmoplast. Phragmoplast serves as a guide for cell plate assembly and the subsequent formation of new cell wall separating two daughter cells (Lee and Liu, 2013).

Complex analyses of cell division and elongation patterns during primary root growth and development were mostly performed in model plants such as Arabidopsis (e.g., Beemster and Baskin, 1998; Beemster et al., 2002; Lavrekha et al., 2017), maize (e.g., Sacks et al., 1997), barley (e.g., Kirschner et al., 2017), tobacco (e.g., Pasternak et al., 2017) and rice (e.g., Rebouillat et al., 2009; Ni et al., 2014). These studies were mostly based on 3D reconstruction analyses from fixed plant samples. Functional studies on dynamics of microtubules during mitotic cell division were almost exclusively conducted in two model species, Arabidopsis and tobacco. The microtubule cytoskeleton in living plant cells can be visualized using several microtubule-specific molecular reporters. One such reporter is the green fluorescent protein (GFP) fusion with the microtubule binding domain (MBD) of the mammalian microtubule-associated protein 4 (MAP4; GFP-MBD). Dynamics, reorientation, and reorganization of cortical microtubules with GFP-MBD as a molecular microtubule marker was originally studied using laser scanning confocal microscope in living epidermal cells (Marc et al., 1998).

Nowadays there are several microscopic approaches to study microtubules in living plant cells of model plant species during cell division. Confocal laser scanning microscopy (CLSM) and spinning disk (SD) microscopy are most popular ones. From practical point of view, dimensions of tobacco BY-2 and Arabidopsis suspension cells, as well as easily cultured Arabidopsis seedling plants facilitate utilization of conventional microscopy methods for live-cell imaging of mitotic microtubules in dividing cells. Another important aspect of microscopic cell division studies on model plants is availability of firmly established transformation protocols and protein tagging approaches in Arabidopsis and tobacco. Some of these studies analyzed stem cell divisions (Campilho et al., 2006) and determined the role of MAP65-1 phosphorylation in cell cycle progression (Boruc et al., 2017) in the Arabidopsis root, visualized Arabidopsis zygote cell divisions (Kimata et al., 2016), described microtubule arrays during cell division of Arabidopsis suspension cells (Calder et al., 2015), or visualized preprophase band (Dhonukshe and Gadella, 2003), mitotic spindle (Dhonukshe et al., 2006; Gaillard et al., 2008), and phragmoplast (Murata et al., 2013) in dividing tobacco BY-2 cells. Further, Arabidopsis γ-tubulin double mutants *tubg1-1* and *tubg2-1* expressing GFP-MBD microtubule marker showed abnormal spindles, asymmetric phragmoplasts, and disorganization of cortical microtubule arrays (Pastuglia et al., 2006). Time-lapse live cell imaging using CLSM was used to document abnormalities of mitotic microtubule arrays and to monitor the duration of mitosis in *anp2anp3* and *mpk4* mutants (Beck et al., 2011). Arabidopsis *fh1-1* mutant expressing GFP-MBD showed increased instability of microtubules (Rosero et al., 2016). SD microscopy was used mostly to study dynamics of cortical microtubule arrays. Bundling of cortical microtubules and speed of microtubule growth and shortening on plus and minus ends were studied in living hypocotyl cells of Arabidopsis (Shaw and Lucas, 2011) while dynamic microtubule nucleation of cortical arrays has been done in Arabidopsis wild type, *katanin* and *gcp2-1* mutants (Nakamura et al., 2010).

Nevertheless, environmental and long-term imaging are compromised in such conventional microscopic methods as CLSM and SD because living model plants are exposed to several stresses (artificial horizontal position, tight microscopic chamber with limited nutrients, and pressure between microscopic slides) and suffer from high energy illumination causing undesirable phototoxicity and photodamage. In comparison to Arabidopsis, crop species including alfalfa typically have more robust habitus and their roots are thicker because they consist from more tissue layers. Consequently, mounting of the whole alfalfa seedlings to classical horizontally-positioned microscopy stage is more challenging and affects fitness of such samples during long-term experiments. Obviously, microscopic techniques like CLSM and SD also suffer from serious limitations in imaging depth. Finally, all commercial CLSM and SD platforms are based on horizontal stages, not allowing for living plants to be observed normally oriented with respect to the gravity vector. All these disadvantages can be avoided by using light-sheet fluorescence microscopy (LSFM). Optical sectioning of the sample with excitation sheet of light is very fast. Importantly, excitation of fluorophores is restricted within the light-sheet volume, which effectively eliminates out-of-focus fluorescence and bleaching. Emitted fluorescence light is detected by independent orthogonally-positioned detection objective. Focal plane of the light-sheet is harmonized with the focal plane of the detection objective and thus, very thin layer of the sample is detected by spherical aberration-free imaging. Sample can be freely rotated with respect to the light-sheet plane illumination allowing multi-angular image acquisition. Fast imaging mode of LSFM ensures that imaged cells are illuminated with very low level of excitation energy (Stelzer, 2015). Finally, in some custom-made and commercial LSFM platforms, sample loading can be done vertically allowing typical gravitropic growth of plants during the course of imaging. All these technical benefits bring LSFM to the forefront of current standards for proper spatial, temporal and physiological imaging of developmental processes. So far the applications of LSFM in plants have been preferentially restricted to the developmental imaging of primary or lateral roots in Arabidopsis (Maizel et al., 2011; Lucas et al., 2013; Rosquete et al., 2013; Vermeer et al., 2014; Ovečka et al., 2015; von Wangenheim et al., 2016). Subcellular localizations of cytoskeletal components by LSFM in Arabidopsis are very few, and include structure and dynamics of both microtubule and actin cytoskeleton in different organs (Ovečka et al., 2015), cell division in root epidermis (Maizel et al., 2011) and developmental imaging of EB1c in cells of different tissues and developmental zones of the root tip (Novák et al., 2016).

Generally, studies on cellular distributions of fluorescently-labeled proteins in *M. sativa* are more difficult due to the limited availability of established molecular tools and efficient transformation techniques. In contrast to Arabidopsis and tobacco, there are only scarce reports on cytoskeletal dynamics during cell division in *Medicago sativa*. It is known that transitions between G1/S and G2/M phases are controlled by cyclin-dependent Ser/Thr kinases, among others also by *M. sativa* kinase CDKB2;1 (Joubès et al., 2000). This kinase is activated in mitotic cells and it is localized in PPB, perinuclear ring in early prophase, mitotic spindle, and phragmoplast (Mészáros et al., 2000). Activity of CDKB2;1 is controlled by protein phosphatase PP2A which affects microtubule organization during the mitosis (Ayaydin et al., 2000). However, both organization and dynamics of microtubules was characterized only in developing and Nod factor-treated root hairs of *Medicago truncatula* (Sieberer et al., 2002, 2005; Timmers et al., 2007). In addition, actin cytoskeleton was visualized in interface and post-mitotic root cells and in root hairs using Plastin-GFP and GFP-mTalin in *M. truncatula* (Voigt et al., 2005). Here we employed for the first time LSFM for long-term live cell imaging of GFP-tagged microtubules in thicker roots of a legume crop plant. We performed quantitative study of root growth rate and duration of individual mitotic stages in diverse tissues of robust roots of transgenic *M. sativa* plants originating from somatic embryogenesis that carried GFP-MBD microtubule marker. We show positive correlation between root growth rate and dynamic patterns of microtubule arrays in dividing cells during continuous root development.

## Materials and methods

### Stable transformation of *Medicago sativa*

*M. sativa* stable transformation has been performed with *Agrobacterium tumefaciens* strain GV3101 containing construct carrying microtubule-binding domain (MBD) of the mammalian MICROTUBULE-ASSOCIATED PROTEIN 4 (MAP4) fused to GFP driven by constitutive 35S promoter. N-terminal GFP fusion with rifampicin and kanamycin resistance was prepared by classical cloning method in pCB302 vector with herbicide phosphinothricin as the selection marker *in planta*. Phosphinothricin has been added to each subculture medium to control successful transformation. The transformation procedure was performed according to protocol for efficient transformation of alfalfa described by Samac and Austin-Phillips (2006). Highly responsive cultivar Regen SY (RSY; Bingham, 1991) of *M. sativa* has been selected. Well-developed leaves at the third node from the shoot apex were used as a source plant material. Leaves explanted from plants were surface sterilized, cut in half and wounded on the surface with sterile scalpel blade. They were incubated with overnight grown *A. tumefaciens* culture with cell density between 0.6 and 0.8 at A_600_ nm for 30 min. Formation of calli and somatic embryos as well as induction and development of shoots and roots were achieved by transferring culture on the appropriate media (Schenk and Hildebrant medium for *A. tumefaciens* inoculation, Gamborg medium for cocultivation, callus initiation and embryo formation and Murashige and Skoog media for plant rooting, development and maintenance) for appropriate time in the culture chamber at 22°C, 50% humidity and 16/8 h light/dark photoperiod (Samac and Austin-Phillips, 2006). Regenerated plants were maintained on media with selective phosphinothricin marker and tested for the presence of GFP fusion proteins using fluorescence microscope. Transgenic *M. sativa* plants were further clonally propagated *in vitro* via somatic embryogenesis from leaf explants. Received somatic embryos stably expressing *35S::GFP:MBD* construct were used in further experiments.

### Plant material and sample preparation for light-sheet microscopy imaging

Transgenic somatic embryos carrying *35S::GFP:MBD* construct with well-developed root poles were separated, individually transferred and inserted vertically into solidified Murashige and Skoog-based root and plant development medium (MMS) or into Murashige and Skoog plant maintaining medium (MS). Embryos were enclosed from the upper part by fluorinated ethylene propylene (FEP) tube with an inner diameter of 4.2 mm and wall thickness of 0.2 mm (Wolf-Technik, Germany). FEP tubes were inserted into the culture medium carefully to enclose individual somatic embryo and surrounding medium inside of the tube. After 1–2 days of somatic embryo stabilization and after visual inspection of the root pole which was starting elongation, such FEP tube with somatic embryo was carefully removed from culture medium and prepared for light-sheet microscopy. Sample was prepared according to the “open system” protocol for long-term live-cell imaging of *Arabidopsis thaliana* seedlings described by Ovečka et al. (2015), with a modification in the respect that *M. sativa* roots are thicker than *A. thaliana* roots. The block of the culture medium inside of the FEP tube serves as holder of the sample and simultaneously also as root growing medium. Upper green part of the somatic embryo and later on developing plantlet was in open space of the FEP tube with the access to air. Thus, the root was able to grow inside of the microscope chamber without stress during long-term experiments. After removing from the culture plate FEP tube with the sample was inserted into sterile plastic syringe (with 1 ml volume and an inner diameter of ~5 mm), which was cut-open at the tip before use. Plastic syringe was used for fixing the sample in sample holder of the microscope. Sample holder with the sample was placed into observation chamber of the microscope tempered to 22°C using Peltier heating/cooling system. Before insertion of the sample to the microscope somatic embryo in the solidified culture medium was ejected slightly out of the FEP tube to the extent that root in the block of solidified culture medium was directly loaded into appropriate liquid medium (MMS or MS) in the microscope chamber. FEP tube was still holding the sample, but was no more surrounding the root part of the plant, which enhanced considerably quality of root deep imaging. To prevent contamination during long-term imaging, liquid medium filling the observation chamber was filter-sterilized using a sterile syringe filter. FEP tube was sterilized using 70% ethanol and dried out before use. After insertion of the sample to the observation chamber of the light-sheet microscope and 30 min stabilization, it was prepared for imaging.

### Light-sheet microscopy

Long-term fluorescence live-cell imaging was done with the light-sheet Z.1 system (Carl Zeiss, Germany), equipped with W Plan-Apochromat 20×/1.0 NA water immersion detection objective (Carl Zeiss, Germany) and two LSFM 10x/0.2 NA illumination objectives (Carl Zeiss, Germany). Samples were imaged using dual-side illumination by a light-sheet modulated with a pivot scan mode. GFP-MBD was visualized with excitation line 488 nm and with emission filter BP505-545. Laser excitation intensity did not exceed 3% of the laser intensity range available. Image acquisition was done every 5 min in Z-stack mode for a period of 3–12 h. Scaling of images in x, y, and z dimensions was 0.152 × 0.152 × 0.469 μm. Images were scanned in two or three views coordinated to follow each other in y axis to record root growth in long-term imaging and to prevent the movement of the growing root out of the field of only one field of view. Images were recorded with the PCO.Edge camera (PCO AG, Germany) with the exposure time 20 or 50 ms per optical section.

### Measurements and statistical analyses

Time-lapsed images of growing roots in x-, y-, z-, and t-dimensions were acquired using Zen 2014 software, black edition (Carl Zeiss, Germany). Images in the czi format were transformed to 2D images using Maximum intensity projection function. Subsets of data containing individual dividing cells were created either with defined x- and y- dimensions from maximum intensity projection images or from original data with defined x-, y-, and z- dimensions. Root growth rate and displacement of individual cells in growing roots (representing a displacement distance of dividing cell in growing root in respect to the growth medium, Baskin, 2013) were recorded from the stage of mitotic spindle formation till the phragmoplast disappearance as well as width of PPB were measured using Line function of the Zen 2014 software. Duration of individual mitotic stages in min (stages of PPB, spindle and phragmoplast) in dividing cells was measured using Coordinate function of the Zen 2014 software. The initial stage of the measurement started at a progressive PPB narrowing which was defined by PPB width when it was in the range of 2–4 μm.

For measurement of duration of mitotic and cytokinetic stages only transverse cell divisions were selected. All parameters were measured and evaluated separately for epidermis and the first outer layer of cortex. Data from 9 individual roots were collected. After quantitative evaluation, measured roots were divided into three groups according to the root growth rate.

Final statistical data evaluation and plotting were done with Microsoft Excel software. Statistical significance was carried out using program STATISTICA 12 (StatSoft) according to one-way ANOVA and subsequent Fisher's LSD test at the level of significance of *P* < 0.05.

## Results

### Germination of *M. sativa* somatic embryos

Light-sheet microscopy imaging was performed on roots of transgenic *M. sativa* plants originating from somatic embryos. After completion of somatic embryogenesis and after maturation, further development of somatic embryos required “germination,” characterized by proliferation on the root pole. This was initiated by transfer of mature somatic embryos from a hormone-free to a root and plant development (MMS) inducing medium. Somatic embryos with already proliferating roots were transferred directly to plant maintaining (MS) medium. Each somatic embryo has been transferred to MMS (or MS) medium individually in such way so that the root pole of somatic embryo was carefully inserted vertically into the solidified culture medium. This ensured that further growth of the root would continue inside of the medium and in vertical orientation. Upon activation of root growth as defined by its visible elongation, such “germinating” somatic embryos were prepared for light-sheet microscopy using FEP tubes.

### Root growth rate

**Figure 1:**
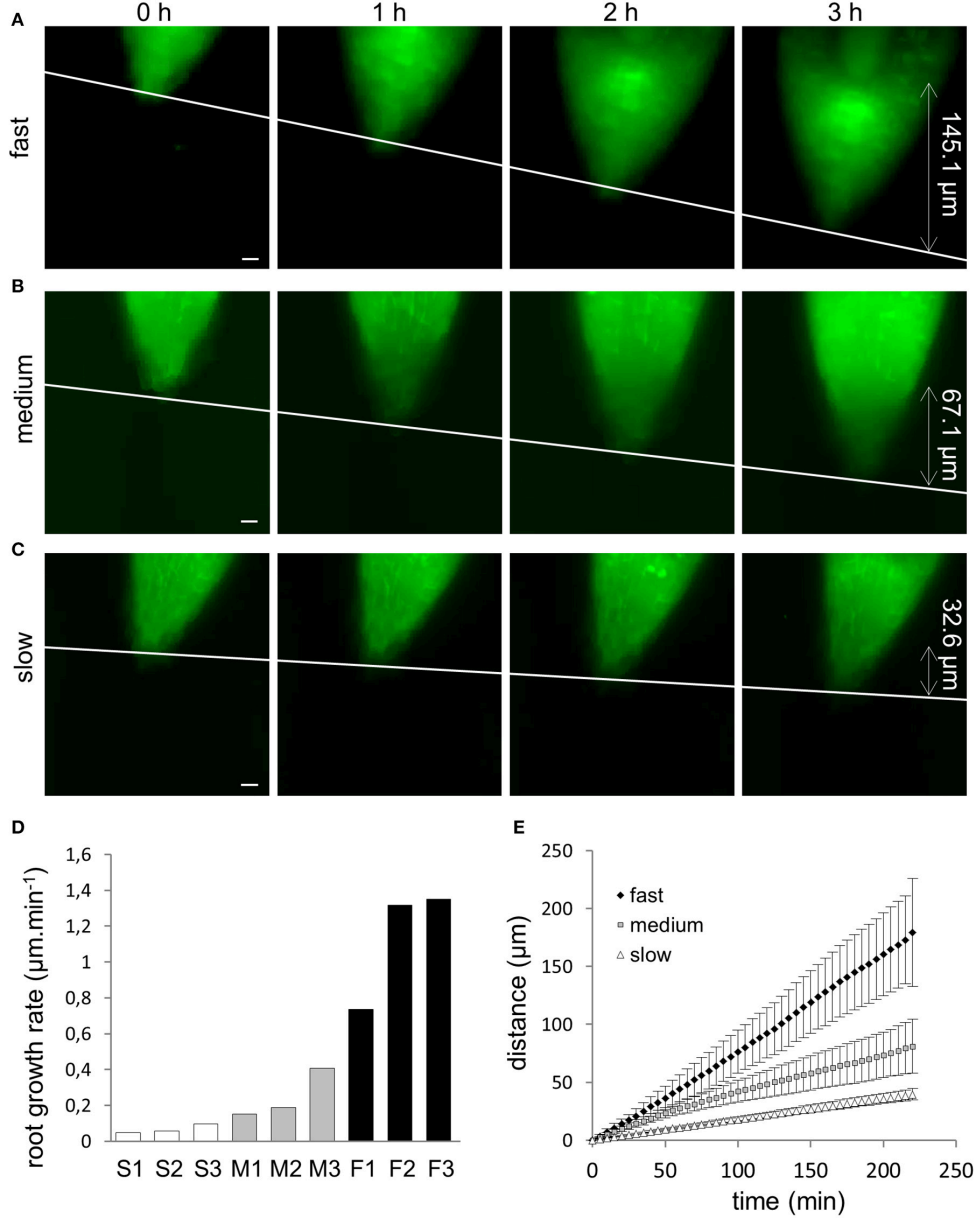
Root growth rate of *Medicago sativa* somatic embryo-derived plantlets, expressing *35S::GFP:MBD* construct. **(A–C)** Light-sheet microscopy imaging of root growth over the period of 3 h. Based on the root growth rate, roots were sub-divided into three different groups. Representative examples of a fast-growing root, grown in a distance of 145.1 μm in 3 h **(A)**, a medium-growing root, grown in a distance of 67.1 μm in 3 h **(B)** and a slow-growing root, grown in a distance of 32.6 μm in 3 h **(C)**. Double-pointed arrows with appropriate values on the right side of images show recorded distance in μm. White line indicates displacement of the root tip in individual time points of the measurement. **(D)** Bar chart illustrating distribution of measured individual roots according to their root growth rate. White bars represent slow-growing roots (S1, S2, S3), gray bars represent medium-growing roots (M1, M2, M3) and black bars represent fast-growing roots (F1, F2, F3). **(E)** Time course of primary root growth showing a correlation between average length increase of fast-growing (♦), medium-growing (□) and slow-growing (Δ) roots and recording time from time-lapse light-sheet imaging at 5 min intervals. Bars = 20 μm **(A–C)**.

Whole plantlets growing in a cylinder of solidified culture medium inside of FEP tubes were placed to the light-sheet microscope. However, due to the bulkiness of the samples, only the apical part of the root was selected for time-lapse imaging. Root growth inside of the light-sheet microscope was recorded in 5 min intervals. Due to the displacement of the continuously growing root tip out of the selected field of view, images were recorded in two or three axially overlapping fields of view. Each field of view was 292.46 × 292.46 μm and thus, recording of root growth in two successive fields of view provided a frame 580 μm long within which we were able to measure root elongation. Based on the variability among characterized roots, their elongation speed was measured over time periods ranging from 3 to 12 h. Most likely due to the somaclonal variability during somatic embryogenesis, nine individual root tips which were taken into account elongated at different extents and so they were classified into three different groups (Figures 1A–D) according to their root growth rate. Roots showing root growth rate in the range between 0.7 and 1.4 μm.min^−1^ were characterized as fast-growing roots (Figures 1A,D,E; Video S1), roots with growth rate in the range of 0.2–0.5 μm.min^−1^ were characterized as medium-growing roots (Figures 1B,D,E; Video S2) and roots with growth rate <0.2 μm.min^−1^ were characterized as slow-growing roots (Figures 1C–E; Video S3). Measurement of the growth distance of representative roots from each group over a period of 3 h during light-sheet microscopy imaging yielded values ranging between 145.1 μm for a fast-growing root (Figure 1A) to 67.1 μm for a medium-growing root (Figure 1B) and 32.6 μm for a slow-growing root (Figure 1C). Selection of individual roots according to their root growth rate (Figure 1D) was taken into consideration in all further quantitative analyses. Analysis of correlation between increase in length of growing roots and a given recording time during time-lapse light-sheet imaging clearly revealed different root growth rate of fast-growing, medium-growing, and slow-growing roots (Figure 1E), giving proof for distribution of analyzed roots into three different groups for further quantitative analyses.

### Determination of cell division stages

The aim of our study was analysis of dynamic properties of microtubule arrays during cell division in root apical meristem, a tissue exhibiting active and coordinated mitotic activity. To visualize microtubule arrays during individual cell division stages in intact transgenic *M. sativa* plants originating from somatic embryos, we performed subcellular localization of MBD tagged with GFP. Progression of cells through the cell division was analyzed on the basis of quantification of the duration of individual cell division stages. Particular transition points characterizing the duration of individual cell division stages (Figure S1) were identified according to Smertenko et al. (2017). The initial stage of the measurement in preprophase and prophase, when progressive PPB narrowing occurs, was defined from the point when width of the PPB was in the range of 2–4 μm (Figure S1a). Transition from preprophase and prophase stage of cell division to mitotic division (prometaphase stage) was defined by the observation of PPB remnants and early mitotic spindle formation (Figure S1b). PPB stage duration was thus defined from PPB narrowing at late G2 phase (Figure S1a) to PPB disappearance at prometaphase (Figure S1b). Stage of mitotic spindle lasted from PPB disappearance and early mitotic spindle formation during prometaphase and finished by last stage of mitotic spindle presence at telophase (Figure S1c). After disappearance of the mitotic spindle further stages during cytokinesis were formations of early (disk) and late (ring and discontinuous) phragmoplasts (Figures S1d,e). For quantitative analysis, both early and late phragmoplast stages together were considered as one common stage (phragmoplast stage).

**Figure 2:**
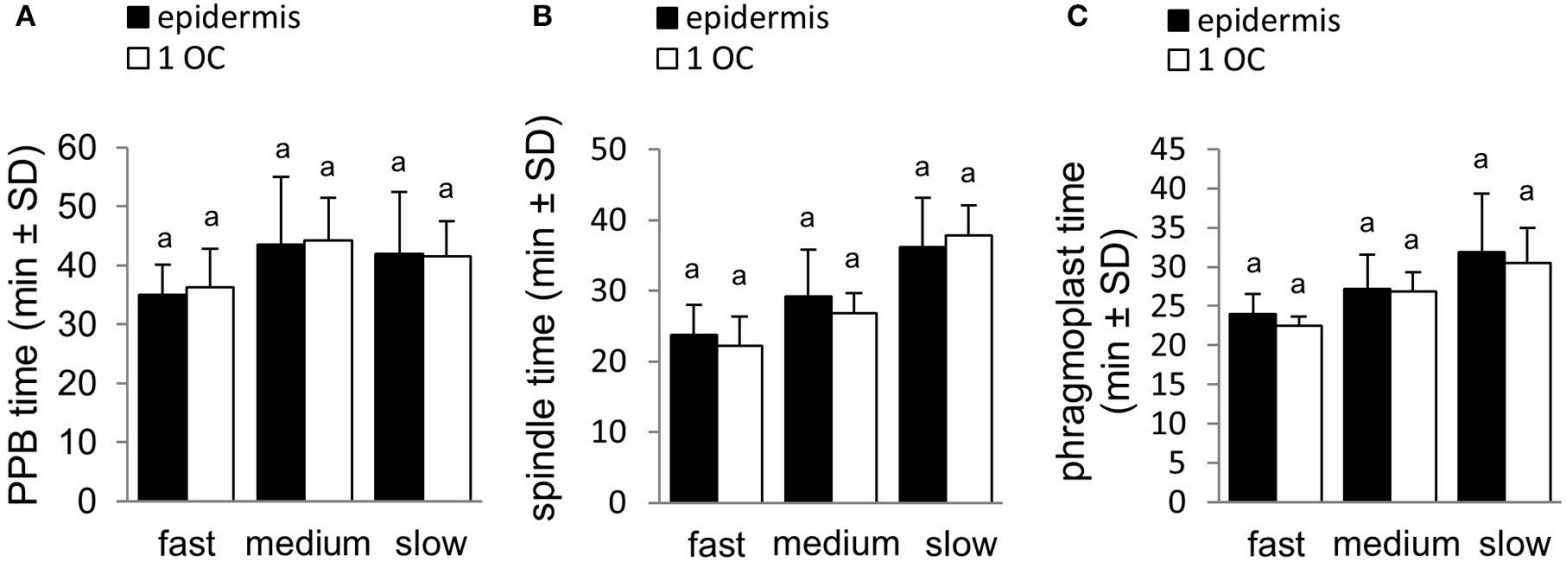
Comparison of duration time of individual cell division stages between epidermis and first outer cortex (1 OC) cell layer. **(A)** Average PPB stage duration in the preprophase and prophase stages of cell division in epidermis and first outer cortex for fast-growing, medium-growing and slow-growing roots. **(B)** Average mitotic spindle stage duration from prometaphase to telophase during cell division in epidermis and first outer cortex for fast-growing, medium-growing and slow-growing roots. **(C)** Average phragmoplast stage duration in cytokinesis of the cell division in epidermis and first outer cortex for fast-growing, medium-growing and slow-growing roots. Letters above the bars represent statistical significance according to one-way ANOVA test at *P* < 0.05.

We analyzed cell division in the epidermis and the first outer cell layer of the root cortex of the root meristematic zone. To reveal if there are some differences or not between these two cell layers, we first characterized their behavior during root elongation growth. In addition to measuring the root elongation extend during the recording time, which was used for the calculation of the root growth rate, we measured also the distance, in which selected dividing cells in epidermis and first outer cortex were displaced along with growing roots. Each dividing cell was tracked down during root elongation and the distance of its displacement (in respect to the growth medium) from the point of mitotic spindle formation until the end of cell division (disappearance of late phragmoplast) was recorded. Results (Figure 2, Figure S2) corroborated the data of root growth rate (Figure 1D). Distances in which selected dividing cells were displaced in individual roots showed the same pattern of distribution into three different groups of roots (Figures S2a,b). Similar results were obtained when the rate of displacement of dividing cells in μm.min^−1^ was measured during the recording of root growth (Figures S2c,d). Importantly, displacement of dividing cells (Figures S2a,b) during the recording of root growth showed no differences between epidermal (Figures S2a,c) and first outer cortex cell layer (Figure S2b,d). Comparison of duration time of individual cell division stages showed several differences between fast-, medium- and slow-growing roots. Different times were recorded in PPB stage duration (Figure S3a), mitotic spindle stage duration (Figure S3b) and phragmoplast stage duration (Figure S3c). Consistently with previous results comparing epidermis with first outer cortex layer (Figure S2), also duration time of individual cell division stages showed no considerable differences between epidermis and first outer cortex cell layer (Figure 2). Based on these evidences, data from individual dividing cells in epidermis and first outer cortex were combined together in each root and analyzed together in further quantitative analyses.

### Duration time of individual cell division stages

**Figure 3:**
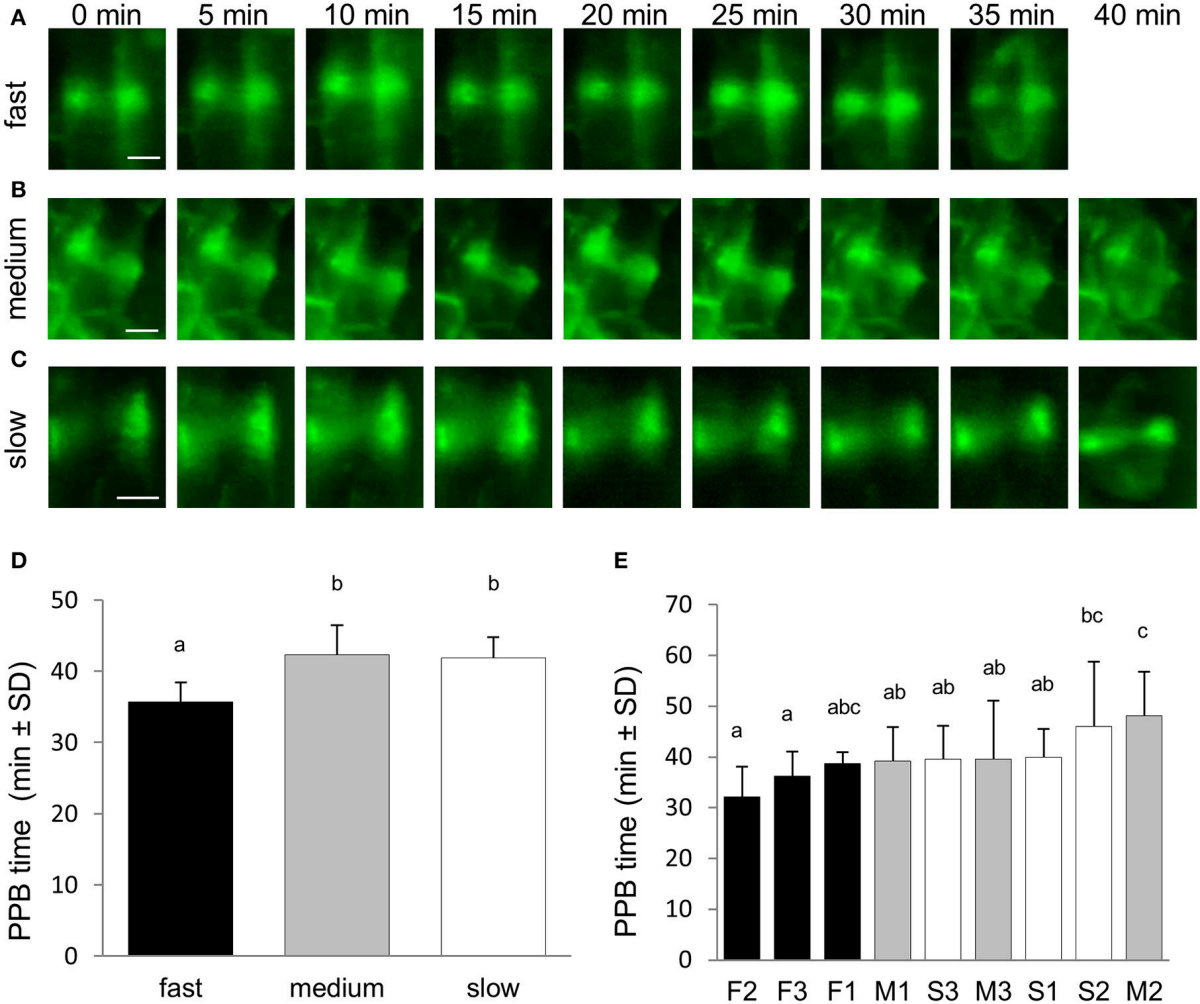
Duration of the preprophase and prophase stage of the cell division. **(A–C)** Series of stills from time-lapse light-sheet imaging of dividing cells in root epidermis and cortex of *M. sativa* plants expressing *35S::GFP:MBD* construct. Sequence of preprophase and prophase stage starting from PPB narrowing and finishing by last stage of PPB presence and early mitotic spindle formation. Progress of the preprophase and prophase stage is shown for fast-growing roots **(A)**, medium-growing roots **(B)**, and slow-growing roots **(C)**. Series of images were recorded in 5 min intervals from the stage of cell division when progressive PPB narrowing was defined by PPB width in the range of 2–4 μm (time 0 min). **(D)** Average duration time of preprophase and prophase stage of cell division in all roots of fast-growing, medium-growing and slow-growing group of roots. **(E)** Average duration time of preprophase and prophase stage in all dividing cells of individual roots. Roots are arranged from shortest to longest average duration time of preprophase and prophase stage. Black bars represent fast-growing roots (F1, F2, F3), gray bars represent medium-growing roots (M1, M2, M3) and white bars represent slow-growing roots (S1, S2, S3). Different letters above the bars represent statistical significance according to one-way ANOVA test at P < 0.05. Bars = 5 μm **(A–C)**.

Time-lapse LSFM imaging of growing roots of transgenic alfalfa plants in 5 min intervals over a period of several hours allowed recording of cells in the meristematic zone and at different stages of cell division. In particular, cells with established PPB (at late G2 phase) were monitored and their entrance to the cell division was documented in time. Preprophase and prophase stage of the cell division was identified from the stage of progressive PPB narrowing when PPB width was in the range of 2–4 μm. This time point has been set up as time 0 min. Recording of preprophase and prophase stage finished by PPB disappearance in cells entering mitosis with starting of mitotic spindle formation in prometaphase. Time-lapse recording of this stage in 5 min intervals revealed its duration for 35–40 min. Interestingly, in roots with different root growth rate this duration was different (Figures 3A–C). In average, preprophase and prophase stage of cell division was fastest in fast-growing roots, while duration of this stage did not differ between medium-growing and slow-growing roots (Figure 3D). Basically the same results were obtained after comparison of preprophase and prophase stage duration in individual roots (Figure 3E).

**Figure 4:**
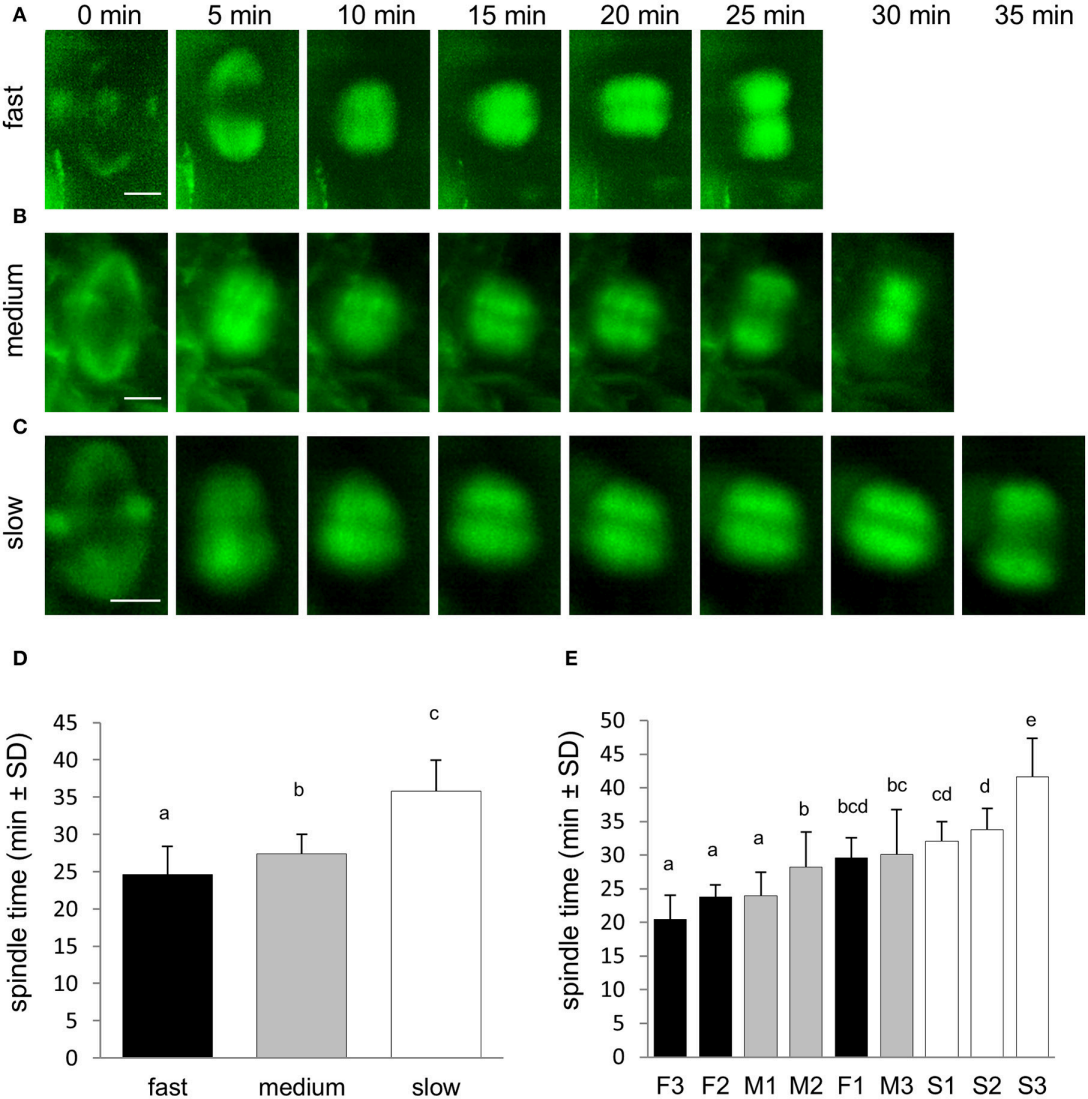
Duration of the mitotic spindle stage from prometaphase to telophase during the cell division. **(A–C)** Series of stills from time-lapse light-sheet imaging of dividing cells in root epidermis and cortex of *M. sativa* plants expressing *35S::GFP:MBD* construct. Sequence of prometaphase, metaphase, anaphase and telophase stages starting from PPB disappearance and mitotic spindle formation at prometaphase and finishing by last stage of mitotic spindle presence at telophase. Progress of the mitotic spindle stages is shown for fast-growing roots **(A)**, medium-growing roots **(B)**, and slow-growing roots **(C)**. Series of images were recorded in 5 min intervals from the PPB disappearance and mitotic spindle formation at prometaphase (time 0 min). **(D)** Average duration time of mitotic spindle (prometaphase, metaphase, anaphase, and telophase) stages of cell division in all roots of fast-growing, medium-growing and slow-growing group of roots. **(E)** Average duration time of mitotic spindle (prometaphase, metaphase, anaphase, and telophase) stages in all dividing cells of individual roots. Roots are arranged from shortest to longest average duration time of mitotic spindle. Black bars represent fast-growing roots (F1, F2, F3), gray bars represent medium-growing roots (M1, M2, M3) and white bars represent slow-growing roots (S1, S2, S3). Different letters above the bars represent statistical significance according to one-way ANOVA test at *P* < 0.05. Bars = 5 μm **(A–C)**.

Stage of mitotic spindle was measured from disappearance of the PPB with simultaneous mitotic spindle formation through metaphase and anaphase to mitotic spindle disappearance at telophase. Measurement revealed that termination of the mitotic spindle stage at telophase was different among three groups of roots differing in their growth rate (Figures 4A–C). The average duration time of this stage revealed that it was shortest in fast-growing roots, significantly longer in medium-growing roots and the longest in slow-growing roots (Figure 4D). Comparison of mitotic spindle duration in individual roots confirmed this observation, showing shortest duration of this stage in fast-growing roots and longest duration in slow-growing roots (Figure 4E).

**Figure 5:**
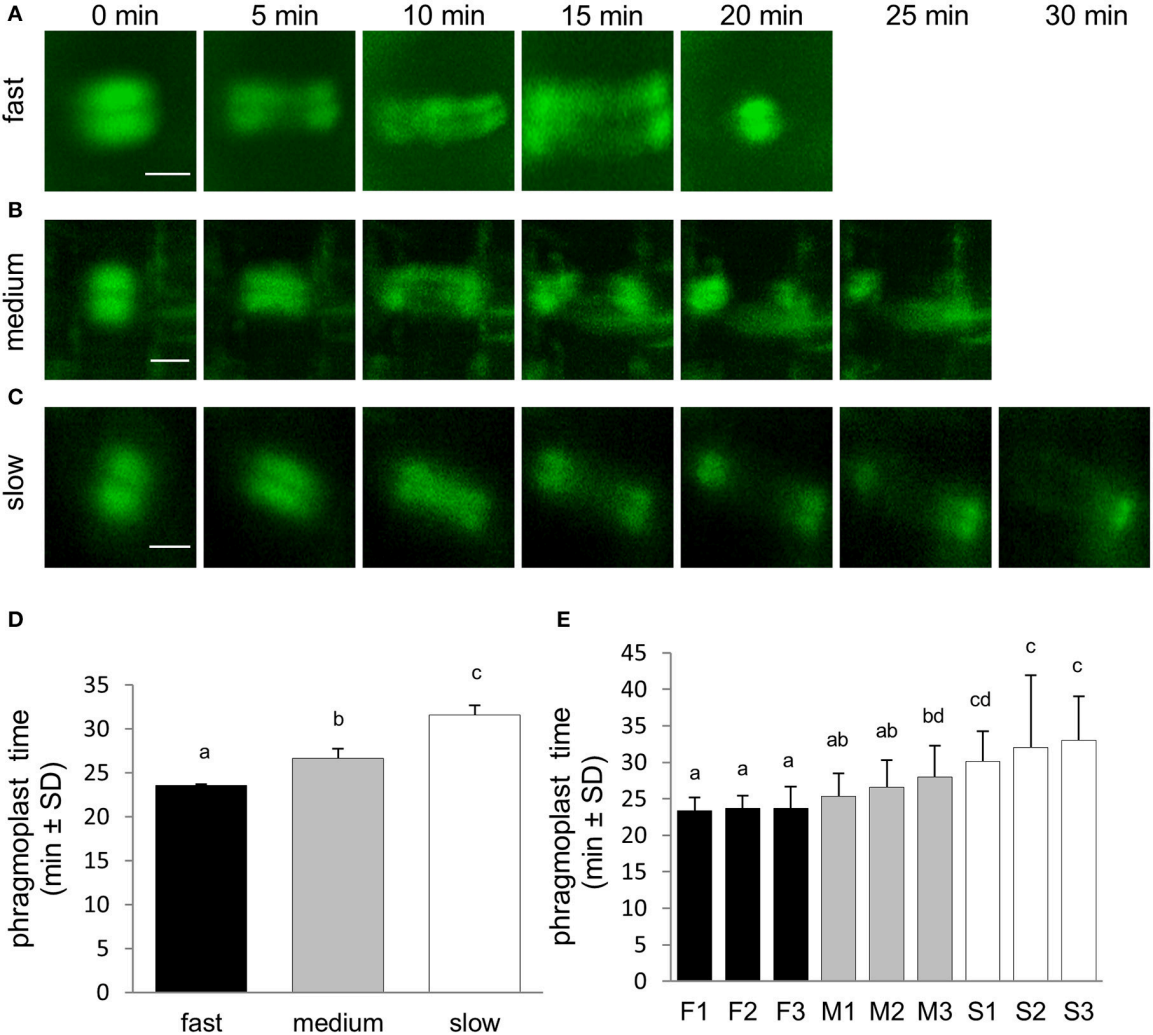
Duration of the phragmoplast stage of the cell division. **(A–C)** Series of stills from time-lapse light-sheet imaging of dividing cells in root epidermis and cortex of *M. sativa* plants expressing *35S::GFP:MBD* construct. Sequence of phragmoplast stages is shown for fast-growing roots **(A)**, medium-growing roots **(B)** and slow-growing roots **(C)**. Series of images were recorded in 5 min intervals from the stage of early phragmoplast formation (time 0 min). **(D)** Average duration time of phragmoplast stage of cell division in all roots of fast-growing, medium-growing, and slow-growing group of roots. **(E)** Average duration time of phragmoplast stage in all dividing cells of individual roots. Roots are arranged from shortest to longest average duration time of phragmoplast. Black bars represent fast-growing roots (F1, F2, F3), gray bars represent medium-growing roots (M1, M2, M3) and white bars represent slow-growing roots (S1, S2, S3). Different letters above the bars represent statistical significance according to one-way ANOVA test at *P* < 0.05. Bars = 5 μm **(A–C)**.

Duration of the phragmoplast stage was determined from mitotic spindle disappearance at telophase with the formation of early (disk) phragmoplast to disappearance of late (ring and discontinuous) phragmoplast at the end of cytokinesis. Consistent with the duration of previous cell division stages the termination of phragmoplast expansion also occurred at different time among the three groups of roots differing in their growth rate (Figures 5A–C). The average duration time of phragmoplast stage was shortest in fast-growing roots, significantly longer in medium-growing roots and the longest in slow-growing roots (Figure 5D). Comparison of phragmoplast stage duration in individual roots confirmed this observation (Figure 5E).

### Correlation between duration of cell division stages and root growth rate

**Figure 6:**
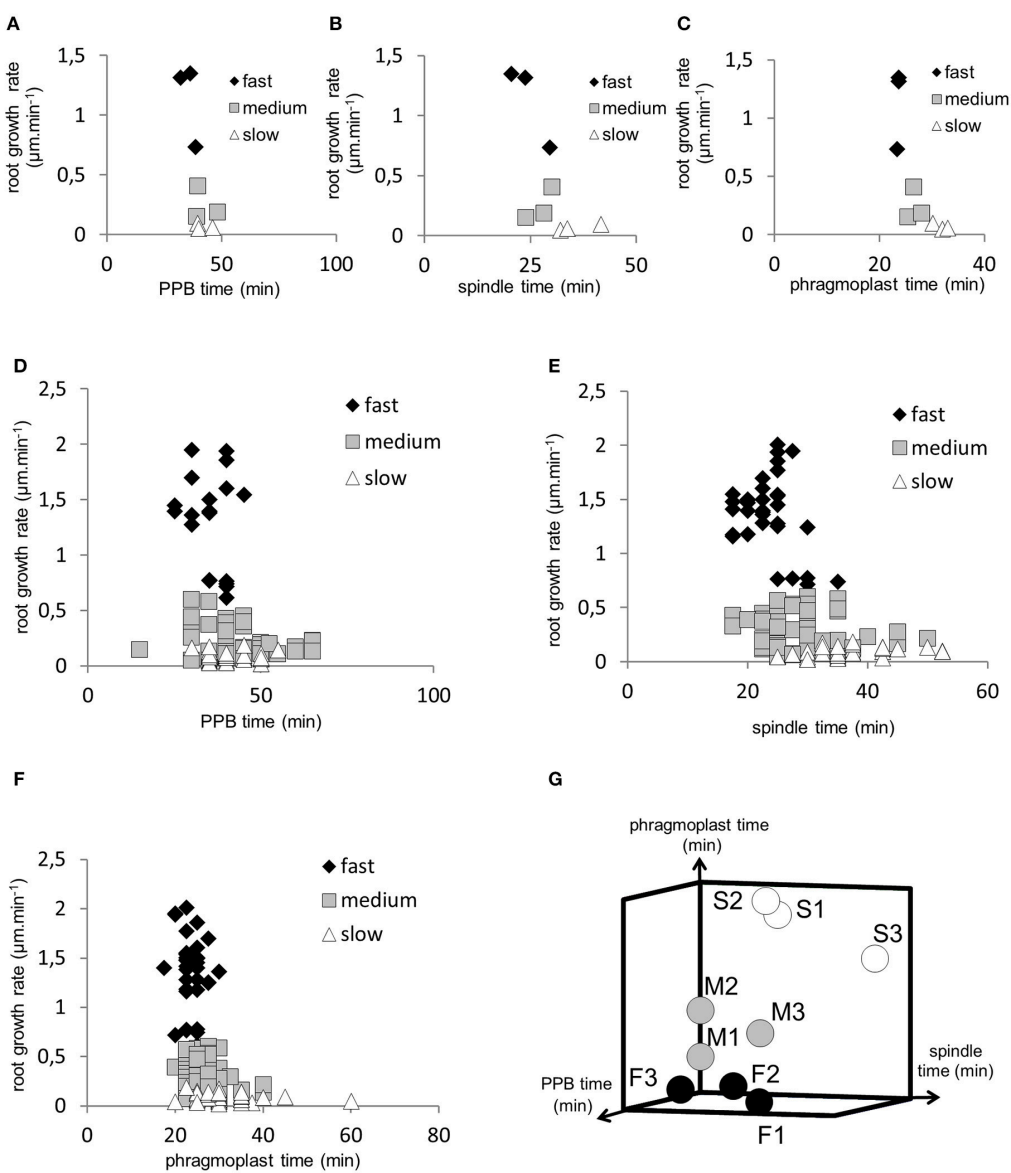
Two- and three-dimensional visualization of data clusters for duration time of individual cell division stages in fast-growing, medium-growing and slow-growing roots according to root growth rate. **(A–C)** Distribution of clusters for average duration time of the PPB stage **(A)**, mitotic spindle stage **(B)** and phragmoplast stage **(C)** according to root growth rate in three fast-growing (♦), three medium-growing (□) and three slow-growing (Δ) roots. **(D–F)** Distribution of clusters for duration time of the PPB stage **(D)**, mitotic spindle stage **(E)** and phragmoplast stage **(F)** according to root growth rate in all individual dividing cells of the fast-growing (♦), medium-growing (□) and slow-growing (Δ) roots. **(G)** Visualization of data clustering for three fast-growing (F1, F2, F3), three medium-growing (M1, M2, M3) and three slow-growing (S1, S2, S3) roots according to cross-correlation between average duration time of the PPB stage, mitotic spindle stage and phragmoplast stage.

**Figure 7:**
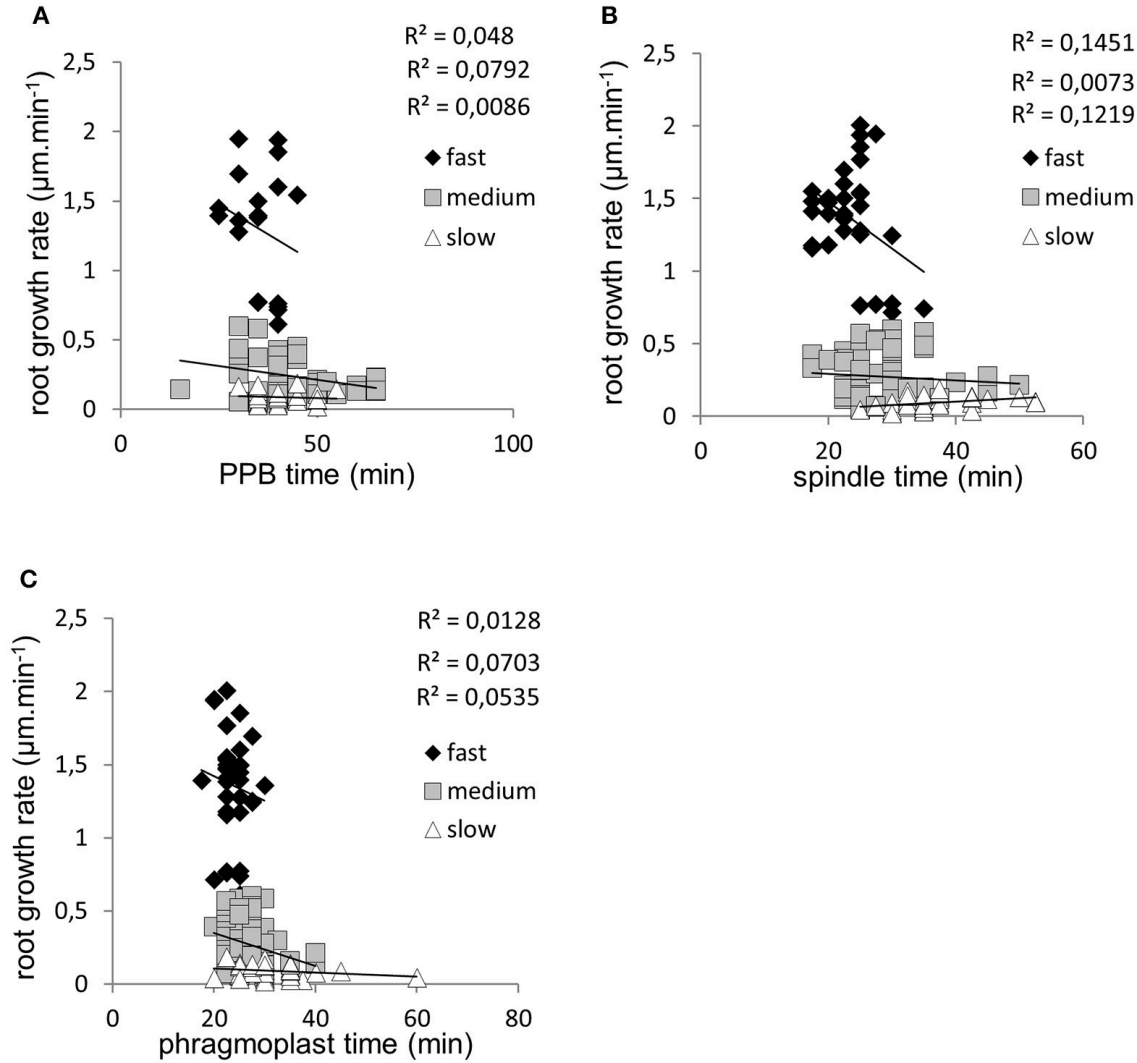
Correlations between root growth rate and duration time of the PPB stage, mitotic spindle stage and phragmoplast stage of dividing cells. **(A)** Distribution of clusters, correlation lines and correlation coefficients for duration time of the PPB stage in all recorded dividing cells of fast-growing (♦), medium-growing (□), and slow-growing (Δ) roots according to root growth rate. **(B)** Distribution of clusters, correlation lines and correlation coefficients for duration time of the mitotic spindle stage in all recorded dividing cells of fast-growing (♦), medium-growing (□) and slow-growing (Δ) roots according to root growth rate. **(C)** Distribution of clusters, correlation lines and correlation coefficients for duration time of the phragmoplast stage in all recorded dividing cells of fast-growing (♦), medium-growing (□), and slow-growing (Δ) roots according to root growth rate.

**Figure 8:**
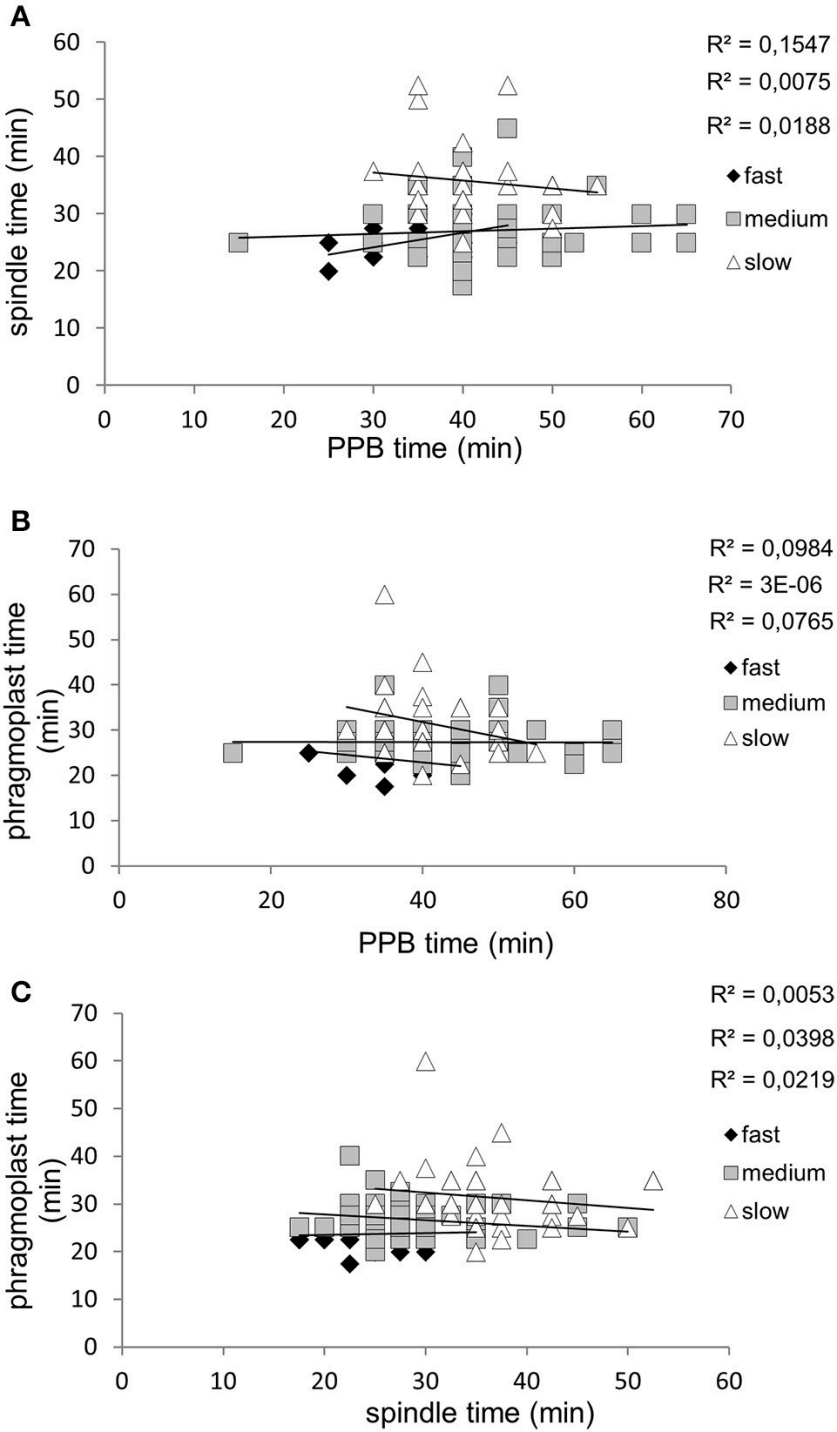
Correlations between duration time of individual cell division stages in all measured dividing cells. **(A)** Correlations between duration time of the PPB stage and mitotic spindle stage in fast-growing (♦), medium-growing (□), and slow-growing (Δ) roots. **(B)** Correlations between duration time of the PPB stage and phragmoplast stage in fast-growing (♦), medium-growing (□) and slow-growing (Δ) roots. **(C)** Correlations between duration time of the mitotic spindle stage and phragmoplast stage in fast-growing (♦), medium-growing (□) and slow-growing (Δ) roots.

To reveal possible relationship between speed of root growth and duration of cell division in root apical meristem we analyzed correlations between duration of individual cell division stages and root growth rates. Three groups of roots with different root growth rates were analyzed separately. Cross-comparison of average root growth rate with average duration of PPB (Figure 6A), mitotic spindle (Figure 6B), and phragmoplast (Figure 6C) stages revealed different tendency. Recorded data were arranged in clusters, distinctly separating groups of fast-growing, medium-growing and slow-growing roots (Figures 6A–C). Overall data from all individual dividing cells showing duration time of the PPB (Figure 6D), mitotic spindle (Figure 6E), and phragmoplast (Figure 6F) stages correlated very well with the root growth rates, and confirmed clear separation of fast-growing roots from the rest. Particularly values for roots F2 and F3 (Figure 1D) segregated separately in the upper part of the graphs (Figures 6D–F). Segregation of values measured in medium- and slow-growing roots, correlating PPB, mitotic spindle, and phragmoplast stages with root growth rates into clusters is also obvious, although less pronounced as for fast-growing roots (Figures 6D–G). In general, the extend of correlation between root growth rate and duration time of the PPB (Figure 6D), mitotic spindle (Figure 6E), and phragmoplast (Figure 6F) stages in all dividing cells of fast-, medium- and slow-growing roots is rather low, as documented by trends of correlation lines and values of correlation coefficients (Figure 7). Degree of correlation is rather low also for duration time of individual cell division stages compared between each other, namely between PPB and mitotic spindle (Figure 8A), PPB and phragmoplast (Figure 8B) and also between mitotic spindle and phragmoplast (Figure 8C). Negative slope of correlation lines (Figure 7) indicates a tendency to prolong cell division stages with reducing root growth rates. Thus, clustering of data recorded from individual cell division stages (Figures 6A–F) and clear separation of these clusters in fast-, medium-, and slow-growing roots according to cross-correlation between average duration time of the PPB, mitotic spindle, and phragmoplast stages (Figure 6G) represents crucial aspect of our quantitative evaluation. Collectively, these results suggest correlative regulation of cell division duration depending on the speed of root growth.

## Discussion

Cell division is key regulatory component of plant morphogenesis and development and one of the most frequently studied biological processes in the plant biology. Monitoring cell divisions in living multicellular plant organs require real time microscopy approach for long-term observation of intact plants that are adapted to proper physiological conditions. Live cell LSFM imaging of dividing cells could ideally resolve details of cytoskeletal division machinery with high temporal resolution. Moreover, four dimensional monitoring of cell division in organ-, tissue-, and cell type-specific context is required for fundamental understanding of cell division regulation during plant growth and development. Nowadays, different microscopy methods are used to study cell division in genetically, developmentally and microscopically-tractable plant model species. Critically important, however, is the adaptation of currently available microscopy techniques for routine live cell developmental imaging of living robust plant samples, such as crop species. Together with application of efficient protocols for stable transformation of crop species, it should be based on implementation of new approaches, tools and concepts enabling scientific progress in plant cell imaging.

Progression of cell division in plant cells is regulated by particular microtubule structures, such as PPB, mitotic spindle, and phragmoplast. These microtubule arrays are involved in cell division plane orientation, symmetry of cell division, segregation of nuclear material into two new cell nuclei and partitioning of cytoplasmic space of dividing cell into two daughter cells. Organization and dynamics of microtubules during individual mitotic and cytokinetic stages control the progression of cell division. Microtubule-dependent functions during cell division are mediated by modulators of microtubule organization and dynamics, such as microtubule-associated proteins (MAPs), End-binding 1 (EB1) proteins, and microtubule severing proteins. Study of MAPs from the MAP65 protein family (Van Damme et al., 2004; Chang et al., 2005; Mao et al., 2005; Smertenko et al., 2006; Ho et al., 2011, 2012) and EB1 proteins (Chan et al., 2003, 2005; Komaki et al., 2009) in their interactions with spindle and phragmoplast microtubules promoted considerably our understanding how microtubules are organized and how their dynamic properties regulate cell division progress and duration. Roles of microtubule-severing proteins were traditionally connected to dynamic reorganization of cortical microtubule arrays. Recent study on knock-out mutant *ktn1-2* revealed that KATANIN 1 contributes to PPB formation and maturation, and is involved in the positioning of the mitotic spindle and the phragmoplast (Komis et al., 2017). An important scientific question will be how complex microtubule network well described in models operates in legume crop species. Starting to address this question in *M. sativa*, we characterized microtubule arrays during the progress of mitotic cell division and sustained root growth. Main motivation of our study was to collect relevant data at near environmental condition, ensuring stress- and artifact-free imaging of living legume plant that is not interfering with normal plant growth and development. This was possible by implementation of LSFM and proper sample preparation protocols into live developmental imaging of alfalfa plants.

Progress of mitotic cell division, distribution of mitoses within the root meristematic zone and cessation of mitotic activity in the root transition zone are tissue-specifically regulated. It has been shown that cells of the cortex and epidermis quit cell division earlier than cells of the endodermis and central cylinder tissues (Baluška et al., 1990; Schmidt et al., 2014). Duration of the PPB stage can be considerably long, as compared to the duration of other subsequent cell division stages during mitosis (Dhonukshe and Gadella, 2003; Vos et al., 2004; Komis et al., 2017). Alterations in the duration of the phragmoplast expansion have been observed in the Arabidopsis *mpk4* mutant, which is devoid of the mitogen activated protein kinase 4. This stage of the cell division was considerably delayed in comparison to control plants (Beck et al., 2011). Herein, we recorded and quantitatively evaluated duration time of PPB, mitotic spindle, and phragmoplast during cell division of epidermal and cortex cells in root meristem of transgenic *M. sativa*. Results suggested that duration time of cell division stages was different in roots with different growth rates. PPB stage was shortest in fast-growing roots, characterized by root growth rate of 0.7–1.4 μm.min^−1^, similarly as duration time of mitotic spindle and phragmoplast. Stages of mitosis and cytokinesis lasted significantly longer time in medium-growing roots with root growth rate of 0.2–0.5 μm.min^−1^ and duration of these stages was the longest in slow-growing roots with growth rate <0.2 μm.min^−1^.

Quantitative kinematic study of primary root growth among 18 different ecotypes of *A. thaliana* revealed considerable variations, functionally linking root growth rate with cell cycle regulation. Along with variation in mature cortical cell length and number of dividing cells, also cell cycle duration contributed considerably to the observed variations in root growth rate (Beemster et al., 2002). Because the rate of cell division and the rate of cell production (defined as number of dividing cells and variation in their cell cycle duration) should be principally distinguished (reviewed by Baskin, 2000), determination of root growth rate ideally combines the spatial profile of cell length, cell velocity, relative elongation rate and cell division rate (Beemster and Baskin, 1998; Baskin, 2000). Functional interconnections of these critical parameters are obvious under unfavorable and stress environmental conditions. Responses of *A. thaliana* roots to temperature changes lead to alterations in the meristem length, which was compensated by changes in the cell length within the elongation zone or by changes in division rate helping to maintain equilibrated cell flux within the root meristem (Yang et al., 2017). Although, it is generally assumed that durations of individual mitotic and cytokinetic stages correlate only indirectly with total cell cycle duration, statistically evaluated parameters in *M. sativa* confirmed *vice versa* correlation of root growth rate with speed of these mitotic and cytokinetic stages in the root meristem. In principle, reduction in root growth rate was reflected by the prolongation of mitotic and cytokinetic stages. Such data were not available in crop species *M. sativa* before.

Moreover, the present study is the first one to provide large-scale imaging and quantitative characterization of cell divisions in the growing robust root of *M. sativa* using developmental live cell imaging. This was achieved by advanced LSFM, providing suitable technological and physiological tool for developmental live cell imaging of dividing cells in plant material with high spatial and excellent temporal resolution. LSFM is mesoscopic imaging method, overcoming several critical problems regarding the proper preparation and long-term maintenance of living plant samples. Low phototoxicity allows monitoring of Arabidopsis root growth for long time under physiological conditions (Maizel et al., 2011; Ovečka et al., 2015). Potential of LSFM for developmental imaging of mitotic microtubule arrays during cell division is obvious, although it was so far utilized only in three studies on Arabidopsis. Previously, cell division in growing primary roots in long-term time-lapse experiments was observed in Arabidopsis plants carrying microtubule molecular markers GFP-MBD (Maizel et al., 2011) or GFP-TUA5 (Ovečka et al., 2015). LSFM was also used for characterization of tissue-specific and developmentally-regulated distribution of nuclear levels of GFP-tagged EB1c protein expressed under the control of native *EB1c* promoter. Quantitative correlation analysis revealed relationship between nuclear size and EB1c expression levels in diverse tissues like epidermis, cortex and endodermis, and in different developmental zones including meristematic, transition and elongation zone of *A. thaliana* roots (Novák et al., 2016). In this study we performed dynamic imaging of mitotic microtubule arrays in robust *M. sativa* growing roots using LSFM. For this purpose, we prepared stably transformed lines of *M. sativa* carrying molecular microtubule marker GFP-MBD and evaluated microtubule organization and dynamics during cell division of both epidermal and cortical root cells. Quantitative evaluation of cell division duration time was based on characterization of individual cell division stages, namely PPB, mitotic spindle and phragmoplast. The study clearly revealed an important link between duration of cell division in the root meristematic cells and root growth rates in *M. sativa*. These results suggest that spatio-temporal organization of microtubule-dependent cell divisions in the root meristem significantly contribute to the root growth rates. Importantly, LSFM is broadly applicable to any genetically modified and fluorescently labeled model legume species such as *M. truncatula* and *Lotus japonicus* as well as to agriculturally important legumes such as soybean, fava bean, pea or chickpea. Moreover, it is potentially interesting also for biotechnological applications such as bioimaging of *M. sativa* lines with RNAi-mediated genetic downregulation of stress-induced mitogen activated protein kinase kinase (SIMKK), an important component of signal transduction cascades in alfalfa (Bekešová et al., 2015), or crop transgenic lines with genetically manipulated cytoskeleton (Komis et al., 2015). Considering big potential of LSFM for deep imaging, it can be adapted and used to study interactions of legume roots expressing diverse subcellular markers (including microtubules) with beneficial microbes such as Rhizobia and mycorrhizal fungi.

Conclusively, previous achievements on Arabidopsis together with data presented in this study demonstrate a high potential of LSFM in developmental live cell imaging of mitotic cytoskeletal arrays in diverse plant species. High temporal and good spatial resolution combined with non-invasive and gentle live-cell imaging of cell divisions during growth and development of *M. sativa* roots favor light-sheet microscopy as the most promising imaging method for the future developmental bioimaging and characterization of robust crop species.

## Author contributions

MO and PV conducted all live cell imaging. PV made all post acquisition analyses and quantitative evaluations with input from MO and JŠ. MO wrote first draft of the manuscript with input from all co-authors. JŠ conceived and supervised study, helped to interpret data, and finalized manuscript.

### Conflict of interest statement

The authors declare that the research was conducted in the absence of any commercial or financial relationships that could be construed as a potential conflict of interest.

## Supplementary material

Video S1 Light-sheet imaging of fast-growing root of *M. sativa* stably expressing a GFP-MBD microtubule marker. Root was recorded for a time period of 3 h. Video was produced from time-lapse image acquisition of every 5 min and is presented in the speed of 10 fps.

Video S2 Light-sheet imaging of medium-growing root of *M. sativa* stably expressing a GFP-MBD microtubule marker. Root was recorded for a time period of 3 h. Video was produced from time-lapse image acquisition of every 5 min and is presented in the speed of 10 fps.

Video S3 Light-sheet imaging of slow-growing root of *M. sativa* stably expressing a GFP-MBD microtubule marker. Root was recorded for a time period of 3 h. Video was produced from time-lapse image acquisition of every 5 min and is presented in the speed of 10 fps.
