# Supplementary material for: Alfalfa Root Growth Rate Correlates with Progression of Microtubules during Mitosis and Cytokinesis as Revealed by Environmental Light-Sheet Microscopy
Source: Front Plant Sci. 2017 Oct 30;8:1870. doi: 10.3389/fpls.2017.01870 (PMC5670501; doi:10.3389/fpls.2017.01870)
Supplement: Supplementary file 4 [file Presentation1.PDF]

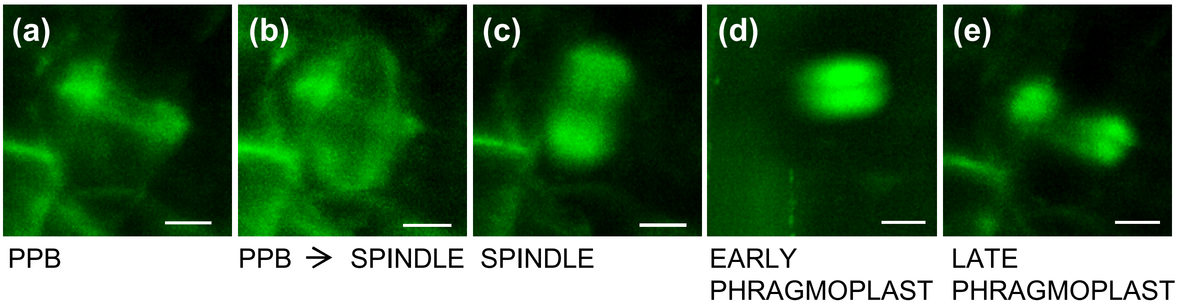

Figure S1. Stages and transition points of cell division in root epidermis and cortex of *M. sativa* plants expressing *35S::GFP:MBD* construct based on time-lapse light-sheet microscopy imaging. (a) PPB narrowing at the pre-prophase stage of cell division. (b) Transition point from pre-prophase to prometaphase of cell division characterized by last PPB presence and early stage of mitotic spindle formation. (c) Typical mitotic spindle stage of cell division (shown is anaphase). (d) Early phragmoplast stage of cell division. (e) Late phragmoplast stage of cell division. Shown is typical example of cell division from medium-growing root. Bars = 5 µm.

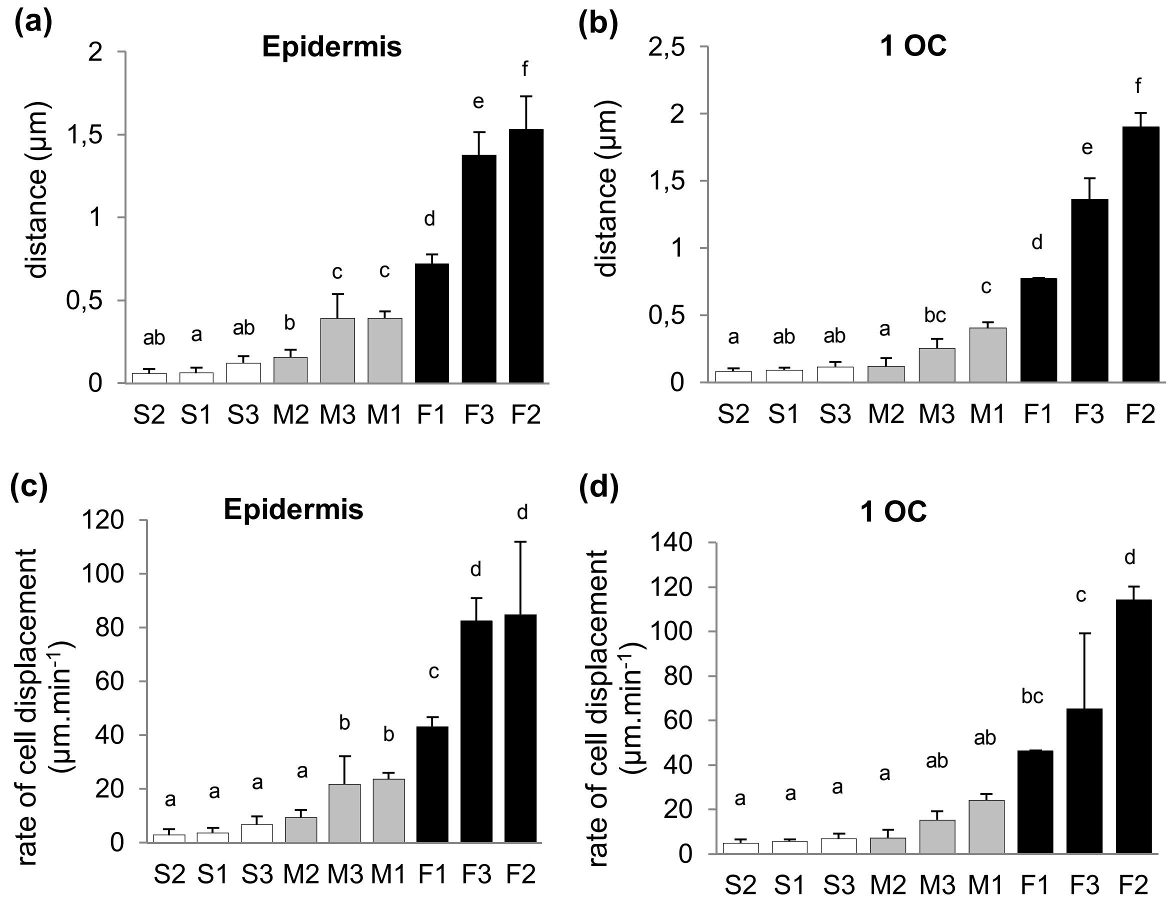

Figure S2. Displacement of dividing cells in epidermis and first outer cortex (1 OC) cell layer during the recording of root growth. (a-b) Average distance, in which dividing cells were displaced in growing roots in respect to growth medium. Selected dividing cells were tracked down from the time of mitotic spindle formation till the end of cell division in root epidermis (a) and in first outer cortex cell layer (b). Data of the measured distances were normalized to the time period of 60 min. (c-d) Average rate of displacement of dividing cells in growing roots in root epidermis (c) and in first outer cortex cell layer (d). Rate of displacement was calculated from distance, in which dividing cells were displaced during the recording of root growth in respect to growth medium and from time required for completion of cell division, starting from mitotic spindle formation till the phragmoplast disappearance. Distribution of measured individual roots is shown from shortest to longest average values for slow-growing (S1, S2, S3, white bars), medium-growing (M1, M2, M3, grey bars) and fast-growing roots (F1, F2, F3, black bars). Different letters above the bars represent statistical significance according to one-way ANOVA test at $P < 0.05$.

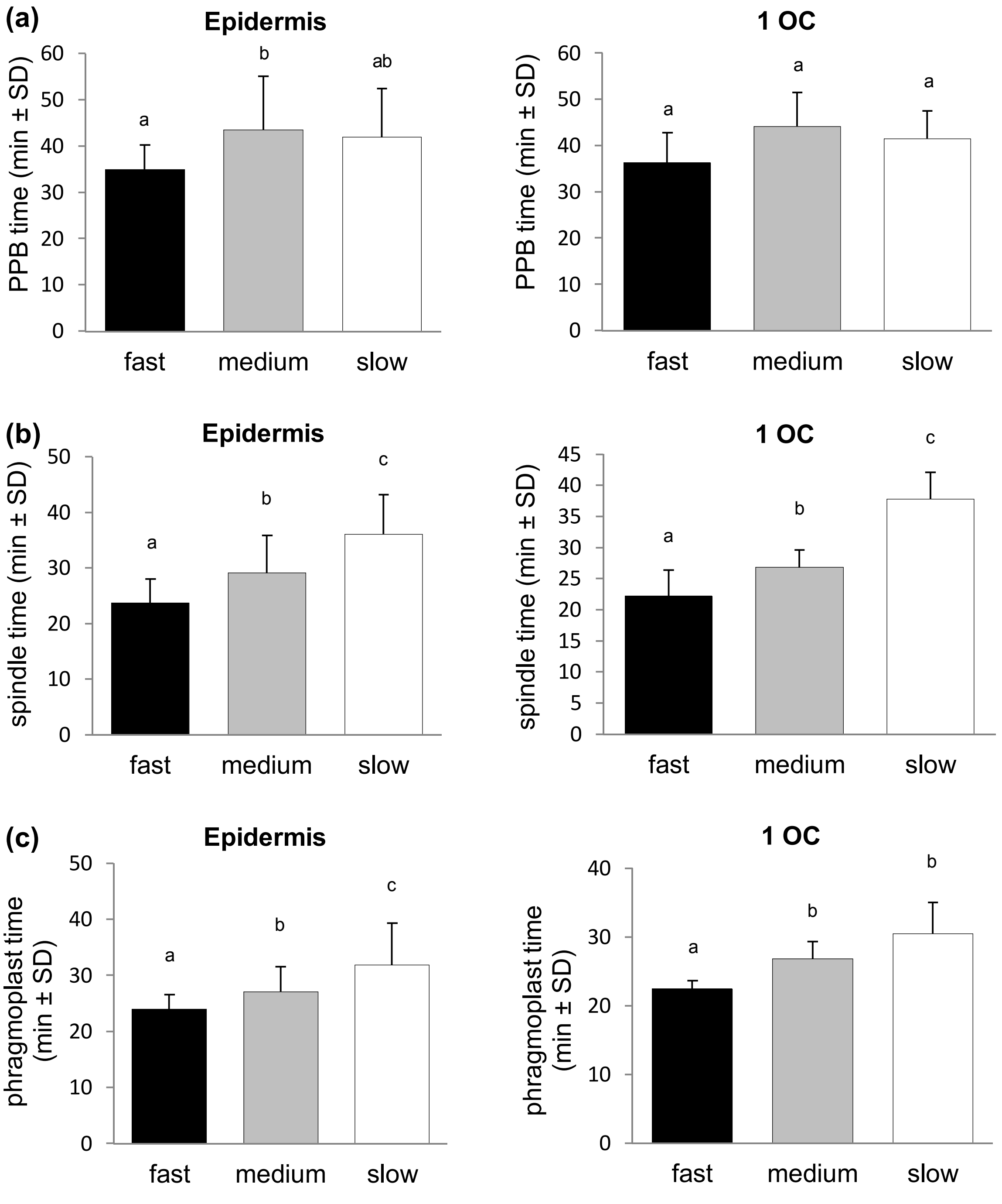

Figure S3. Comparison of duration time of individual cell division stages between epidermis and first outer cortex (1 OC) cell layer. (a) Average PPB stage duration in the pre-prophase and prophase stages of cell division in epidermis and first outer cortex. (b) Average mitotic spindle stage duration from prometaphase to telophase during cell division in epidermis and first outer cortex. (c) Average phragmoplast stage duration in cytokinesis of the cell division in epidermis and first outer cortex. Black bars represent fast-growing roots, grey bars represent medium-growing roots and white bars represent slow-growing roots. Different letters above the bars represent statistical significance according to one-way ANOVA test at $P < 0.05$.
